# Pulmonary Function Tests as a Biomarker in Diffuse Idiopathic Pulmonary Neuroendocrine Cell Hyperplasia Patients Treated With Somatostatin Analogues

**DOI:** 10.7759/cureus.32454

**Published:** 2022-12-12

**Authors:** Heer V Shah, Meer Shah, Krishan Mahathevan

**Affiliations:** 1 Internal Medicine, St George's Hospital, London, GBR; 2 Internal Medicine, Cardiff University, Cardiff, GBR; 3 Acute Medicine, Princess Alexandra Hospital, Harlow, GBR

**Keywords:** diffuse idiopathic pulmonary neuroendocrine cell hyperplasia, biomarker, pulmonary disease, somatostatin analogues, pulmonary function tests

## Abstract

Diffuse idiopathic pulmonary neuroendocrine cell hyperplasia (DIPNECH) describes an indolent, under-recognised and poorly understood pulmonary condition with fewer than 200 reported cases across the literature. Currently, cases are diagnosed following a biopsy of the primary lesion, with treatment options centring on symptomatic benefit as opposed to targeting the underlying aetiology. Classically, DIPNECH lesions have been described as slow growing and benign, but with growing awareness of the condition, reports of metastatic disease with significant symptomatic burden have been reported. However, effectively addressing the subset of DIPNECH patients with greater metastatic potential remains an unmet clinical need.

Due to the similarities between DIPNECH and carcinoid patients, several centres have considered using somatostatin analogues to not only help symptomatically but also to initiate tumour regression. However, to date, there are limited biomarkers to help evaluate the benefit of such options. In this review, we consider the use of pulmonary function tests (PFTs) to help quantify the benefit of somatostatin analogues. Although much of the evidence stems from small single-centre studies, the use of PFTs within the treatment pathway for both localised and metastatic DIPNECH represents a meaningful improvement from subjective monitoring of disease.

## Introduction and background

Characteristics of diffuse idiopathic pulmonary neuroendocrine cell hyperplasia (DIPNECH) 

DIPNECH describes an indolent, exceptionally rare, and inadequately understood pulmonary disorder with under 200 cases across the literature [1]. The condition was first described in 1992, following an open lung biopsy on six non-smoking patients who presented with exertional dyspnoea and cough [2]. The biopsy revealed ‘diffuse hyperplasia and dysplasia of pulmonary neuroendocrine cells, multiple carcinoid tumourlets, and peribronchiolar fibrosis obliterating small airways’ [2]. Due to the absence of any underlying aetiology, the term idiopathic was included within the name.

Histologically, DIPNECH is recognised in a range of forms following a biopsy. It is accepted as a ‘generalised proliferation of scattered neuroendocrine cells, small nodules (neuroendocrine bodies) or a linear proliferation of pulmonary neuroendocrine cells’ [3]. The presence of more dominant nodules may represent proliferations that outspread the basement membrane to form tumourlets (distinct aggregates of neuroendocrine cells <5mm in diameter) or larger carcinoid tumours (nodules >5mm in diameter) [4]. The spectrum of cells described is consistent with DIPNECH’s recognition by the World Health Organisation (WHO) as a preneoplastic lesion [5].

In contrast to extrapulmonary carcinoid tumours where no obvious sex preference has been identified, DIPNECH occurs predominantly in females (9:1) and at a higher mean age of 66 years [1]. While patients are most often non-smokers, occurrences in active smokers have also been recorded [3,6]. Clinically, patients present with symptoms of dyspnoea, chronic non-productive cough and fatigue attributed to frequent misdiagnoses. Two studies in 2015 found no correctly diagnosed cases within their sample, with primary lung cancer, chronic obstructive pulmonary disease (COPD) and bronchiectasis amongst the given diagnoses [1,7].

Whilst DIPNECH remains a solely histological diagnosis, characteristic high-resolution computed tomography (HRCT) findings can aid identification. Radiologically lesions are almost always bilateral where suggestive features of airway disease including bronchial wall thickening and bronchiectasis are commonly observed. Mosaic perfusion is considered the most significant and suggestive feature of DIPNECH with a better appreciation of expiratory CT scans [8]. Additionally, pulmonary function tests (PFTs) and spirometry are useful in gauging the extent of lung function compromise. Pulmonary function in patients with DIPNECH is vastly characterised as obstructive or mixed obstructive/restrictive defects and rarely solely restrictive [9]. Despite the indolent, slow-growing nature of the disease, spirometry in combination with HRCT offers a promising way to closely monitor patients for signs of progression.

## Review

Current treatment options

Current management options are limited and require a multi-disciplinary approach coupled with life-long surveillance. Surgical resections in patients with dominant nodules offer symptomatic relief, whilst also reducing the progression toward severe disease and metastatic spread. Two case reports in 2011 and 2019 both described cases of parenchymal sparing wedge resections in patients with DIPNECH [6,10]. However, in the presence of larger nodules, surgical resection often constitutes lobectomy [6,11,12]. Patients with DIPNECH may occasionally present with debilitating respiratory symptoms and deteriorating pulmonary function. In such cases, a lung transplant has been shown to be a viable and life-saving treatment option [13,14]. 

The lack of licenced, targeted pharmacological therapies for DIPNECH leaves clinicians with treatment options aimed at alleviating symptoms rather than disrupting the underlying aetiology. For instance, patients are given corticosteroids and a combination of short and long-acting beta-agonists to relieve dyspnoea and improve obstructive lung function but evidence on efficacy within this population remains scarce [7,15,16]. Recently, there has been an increased use of experimental therapeutics alongside conventional symptom-based treatments representing the need to find a more targeted solution for DIPNECH. For example, in one small single-centre study, six DIPNECH patients were trialled on azithromycin based on its anti-inflammatory and immunomodulatory effects [17]. All patients within this cohort reported subjective symptomatic relief and reduced cough during their follow-up [18].

Mutations and dysregulation of the mechanistic target of the rapamycin (mTOR) pathway have been well described in conventional neuroendocrine tumours (NETs) [19]. The presence of these aberrant pathways has prompted the use of everolimus which has been shown to significantly improve progression-free survival in patients with progressive lung NETs, leading to FDA approval [19]. A retrospective immunochemistry analysis confirmed an equivalent activation of the mTOR pathway within DIPNECH cells [20]. In light of these findings, three DIPNECH patients with progressive disease who were previously on bronchodilators and corticosteroids were trialled on oral sirolimus alongside their existing treatment. All three patients reported symptomatic improvement of their exertional dyspnoea, as well as radiological improvement, during a 12-month follow-up.

Similarly, the expression of somatostatin receptors (SSTR), particularly SSTR2 is well documented in pulmonary carcinoids advocating the use of somatostatin analogues (SSAs) as a targeted therapy [21,22]. One study demonstrated STTR expression within DIPNECH in 91% of their patients; however, the accumulation seen on OctreoScan was confined to the dominant lesion [23]. In another study of 19 DIPNECH patients who had SSTR imaging, seven patients showed no significant uptake [24]. Likewise, a 2006 case report showed no increased accumulation of^ 111^In-octreotide scintigraphy (an OctreoScan using radiolabeled with indium-111) within the lungs of a DIPNECH patient [12]. Possible reasons for these discrepancies have been attributed to differences in scan sensitivity and the variety in the size of neuroendocrine nodules amongst patients (with larger, more heterogenous nodules showing more uptake) rather than actual differences in SSTR expression [24,25]. This is consistent with evidence confirming SSTR expression increases with greater tumour differentiation [26].

Studies monitoring the effects of SSAs on DIPNECH have described largely positive findings. One retrospective study of five DIPNECH patients started on octreotide identified drastic improvements in cough from four patients [27]. Similarly, eight patients complaining of respiratory symptoms attributed to their DIPNECH were trialled on SSAs, either in combination with their existing therapy or as a monotherapy. At follow-up, these patients stated subjective improvements in their dyspnoea and/or cough [18]. A 2020 study of changes in PFTs in response to SSA treatment also reported symptomatic improvements in 32 patients (76%) receiving SSAs, of which 11 reported significant improvements from their baseline [24]. Likewise, a 2015 study analysing longitudinal clinical data of DIPNECH patients noted improvement in a cough that allowed for increased social activity in three patients (27%) who were started on octreotide [7]. Based on these findings and other studies within the literature, the 2020 National Comprehensive Cancer Network (NCCN) Neuroendocrine Tumour Guidelines recommends SSAs (lanreotide and octreotide) for palliative use in controlling chronic respiratory symptoms in DIPNECH [25].

The increased recognition and understanding of DIPNECH offers an opportunity for innovative therapeutics to continue to be trialled as a means of improving patient prognoses. However, the true efficacy of these therapeutics is often negated due to the subjectivity involved in measuring clinical response. The use of patient-reported outcomes or validated quality-of-life questionnaires have also been advocated, which would provide more objective and replicable metrics [24]. Corroborating any symptomatic improvements within DIPNECH patients with corresponding changes on an objective biomarker would help to optimise the development of therapeutics and improve the validity of any conclusions drawn. For this reason, we propose the use of PFTs, with particular emphasis on spirometry as a validated response biomarker to quantitatively capture improvements in lung function from treatment with SSAs in DIPNECH patients. 

PFTs as a Biomarker

PFTs are a non-invasive set of investigations that quantify lung function. PFTs recognise obstructive and restrictive patterns of lung disease allowing accurate diagnoses and prompting suitable subsequent management. Spirometry is a well-established PFT providing three measurements: volume, time and flow. It is objective, sensitive to early change and reproducible, thus making it an appropriate biomarker for this case study [28].

Spirometry Produces the Following Measurements

Forced expiratory volume in 1 second (FEV1) is the total volume of air breathed out in a second, measured in litres. Forced vital capacity (FVC) is the total volume of air breathed out in one continuous breath, measured in litres. Vital Capacity (VC): The total volume of air breathed whilst at rest, measured in litres. FEV1/FVC ratio is the FEV1 divided by the FVC used in identifying obstructive, restrictive and mixed patterns of lung function. 

The values derived from spirometry are compared to pre-existing data, either reference values or predicted values to allow interpretation. Reference values are formulated from population data consisting of multiple influential variables such as age, ethnicity, smoking status, height, weight, sex, etc. This data is then compiled into equations that generate reference values [29]. Within this essay, the words PFTs and spirometry will be used interchangeably. 

Testing the Validity of Spirometry

Within a given test population there is known heterogeneity amongst exhalations during FVC manoeuvres. A 1982 study best represented the diversity within FVC manoeuvres using 24 standard volume time waveforms [30]. These waveforms are in turn used to power a computer-controlled mechanical syringe that validates the spirometry system. These computer-controlled mechanical syringes should be accurate to within 50 mL for FEV1 and FVC [31]. 

The Discovery of Spirometry

The word spirometer originates from the Latin constituents of spiro (to breathe) and meter (to measure). Evidence of a physical spirometer dates back to a device invented by the British surgeon John Hutchinson in the 1840s. The large device used a calibrated bucket submerged in water which was attached to a breathing tube. Exhaled air in the tube could then be accurately measured [32]. Hutchinson was also responsible for the term vital capacity meaning ‘capacity for life’ indicating the relationship noticed between vital capacity and premature mortality. Despite the clear potential for spirometry use in disease management, accessibility to the required apparatus as well as difficulty in reproducing results delayed its adoption.

Almost a century later, in 1933, German doctor J. Hermannsen first recorded the maximum exhilaration during continuous voluntary effort [33]. This description helped establish a link between the complaint of dyspnoea and maximum breathing capacity (MBC). A decade later, a French study noted that both the rate of breathing and circulating air (vital capacity) both increased during exercise. Using this knowledge, they proposed to measure the largest expired respiratory volume in one second, described as the ‘capacité pulmonaire utilisable à l'effort’ (CPUE), the ‘pulmonary capacity usable on exercise’. Later analysis of the effects of bronchodilator administration on CPUE helped create an index mapping out respiratory disease and their impact on airways [34,35].

Over time, the need to establish homogonous nomenclature led to ‘volume expiratoire maximum (ou maximal) seconde’ (VEMS) replacing CPUE [36]. Finally, in 1957, the British Thoracic Society also reached a consensus on terminology for measurements of respiratory function. The FEV1 was deemed acceptable for clinical purposes and a ratio using either FVC or VC was considered appropriate [37].

An Overview of the Current Uses of Spirometry

Spirometry is used extensively in respiratory medicine. It can be used to aid diagnosis, initiate management, and monitor conditions during follow-up. Primarily, the FEV1/FVC ratio defines the pattern of lung disease. A ratio >0.70 is associated with restricted lung disease, whereas a ratio <0.70 is associated with obstructive lung disease. 

Spirometry in COPD

A post-bronchodilator FEV1/FVC ratio <0.70 is classically considered diagnostic for COPD, a condition characterised by irreversible and progressive airflow resistance [38]. Once an irreversible obstructive lung pattern is noted, FEV1 values are used to stratify patients into GOLD categories indicating the severity of their disease. GOLD-1 implies mild disease with FEV1 ≥80% of predicted, whereas GOLD-4 implies a patient has a severe disease with FEV1 ≥30% of predicted [39]. Whilst, a low FEV1 may indicate increased severity of disease, it does not necessarily correlate with disease activity. For example, an older patient with lower FEV1 and therefore a higher GOLD stage may have a less active disease than a younger patient with higher FEV1 and therefore a lower GOLD stage [38]. Despite this limitation, spirometry remains an essential tool in diagnosing and classifying COPD patients.

A recent literature review analysed the correlation between PFTs and patient-reported outcomes (PROs). These PROs were derived from the Transition Dyspnoea Index (TDI); an indicator of change in dyspnoea from baseline and The St. George’s Respiratory Symptom Questionnaire (SGRQ), which captures information on health-related quality of life and the impact of disease on well-being and activities of daily living. Their results found that patients with greater improvements in trough FEV1 after treatment had better SGRQ and TDI scores, fewer COPD exacerbations and also used less rescue pack medications [40]. Therefore, spirometry and particularly FEV1 improvements correlate well with improvements in PROs and can be used by clinicians to monitor holistic changes during the course of a patient’s disease treatment.

Conversely, numerous studies have highlighted the association between poorer lung function and increased mortality. For example, a study following >1,100 patients over a 29-year period found that predicted FEV1 of normal (FEV1%pred) adjusted to baseline characteristics (age, sex, smoking status, BMI, etc.) was inversely related to all-cause mortality across both males and females [41]. Another similar study analysed whether the GOLD classification of COPD predicts mortality in a cohort of over 15,000 patients. Their findings corroborate with the previous study showing that all GOLD categories, after adjusting for covariates, predicted a higher risk of death [42]. These studies all help to highlight the essential role and comprehensive utility of spirometry and how it has helped revolutionise care within the COPD sphere.

Spirometry in Asthma

Asthma is a chronic respiratory condition with characteristic airway inflammation and hyperresponsivity. Clinically, it presents as a symptom complex of dyspnoea, auditory wheeze, chest tightness and cough with objective evidence of reversible airway obstruction [43]. Spirometry forms an essential part of diagnosis helping establishes postbronchodilator lung function reversibility. It is important to note however that an initial normal lung function does not exclude a diagnosis of asthma [44]. Spirometry is also used within the validated Asthma Control Questionnaire (ACQ) aimed at assessing the adequacy of asthma control [45]. Spirometry has also been used to evaluate the risk of future adverse outcomes in asthma patients, with a low FEV1 labelled as a modifiable independent risk factor for exacerbations [46]. Spirometry, therefore, contributes invaluable data to asthma research and helps clinicians better understand their patients’ prognoses. 

Spirometry and PFTs in DIPNECH

DIPNECH is predominantly characterised by an obstructive pattern of lung disease [4]. Although the risk of metastatic spread of DIPNECH-associated pulmonary NETs is considered rare, cases of extrapulmonary carcinoid tumours including liver metastases have been reported [47]. Metastatic spread develops following the development of pulmonary NETs, necessitating the need for life-long surveillance [25]. In patients who progress down the spectrum from DIPNECH to pulmonary NETs, it is likely that their lung function would deteriorate in proportion to the increased lung tumour volume. Consequently, symptoms of dyspnoea and wheezing would be more frequent and debilitating. As such, we hypothesise that regression in tumour volume would improve both pulmonary function and symptoms.

Supporting this hypothesis, a study measuring the change in PFTs following radiotherapy in non-small-cell lung cancer (NSCLC) patients showed significant improvements in FEV1 after radiotherapy-induced tumour regression [48]. Similarly, a study of 62 patients with inoperable NSCLC compared baseline FEV1 with three-month FEV1 post-radiotherapy. In this study, mean tumour regression was 75% and improvements in FEV1 were best correlated with tumour regression in both central and peripheral tumours [49]. Whilst NSCLC may be more sensitive to radiotherapy, the principles of tumour regression in an inoperable disease improving lung function give hope to potential translatability for the DIPNECH population with multiple inoperable nodules. 

Considering the dispersed nature of DIPNECH nodules found on presentation, targeted radiotherapy may be limited to the largest nodules and therefore offers a partial treatment option. Consequently, alternative therapeutics that can induce homogonous tumour regression should be considered. The antiproliferative properties of SSAs have been demonstrated in large clinical trials and thus offer a viable treatment option for DIPNECH [50]. Hence, monitoring PFTs in DIPNECH patients treated with SSA advances the previously reported findings by utilising a biomarker to corroborate with any symptomatic improvements.

One study initiated 42 DIPNECH patients with SSAs [24]. At baseline, 12 of 15 patients within their sample had pulmonary impairments diagnosed by FEV1% of normal. 93% of patients showed improvements in the PFT value post-treatment. Furthermore, patients with normal PFT values at baseline also showed improvement following SSA therapy. In another study, six patients with progressive DIPNECH defined as an increase in the size and number of lung lesions and/or symptomatic deterioration were treated with SSAs for a mean of 11.3 months. Four of these patients showed an improvement in mean FEV1 (from 50% ± 13% before SSA treatment to 68% ± 17% after SSA treatment at the last follow-up) [23]. Another study in 2020 treated 14 DIPNECH patients with SSAs, of which eight patients had follow-up PFTs [51]. 62.5% of patients had either stable or improved PFTs on follow-up. Moreover, a study treating 11 patients with SSAs showed stabilisation of lung function in all patients with follow-up data [7]. A 2021 retrospective study found PFT improvements in 75% of their sample with no cases of progression to carcinoid tumour following induction of SSA therapy [52]. The studies all advocate the use of SSAs in DIPNECH and showcase the benefits of utilising PFTs to map the success of treatment.

Notable issues

The number of DIPNECH studies using PFTs as a biomarker for measuring response to treatment remains low. Amongst these few studies, several noteworthy issues should be raised. For instance, in two studies, there was a significant decrease in the number of patients for whom follow-up PFT data were available [24,51]. This reduction in sample size poses substantial implications for the validity of any conclusions drawn from the data.

Furthermore, multiple studies have reported data on post-treatment FEV1 without specifying the exact elapsed time after initiating treatment till a repeat FEV1 was measured. Moreover, the frequency of FEV1/PFT testing during follow-up is rarely specified and likely to be highly variable among the studies [7,23,24,52]. This variability reduces the usefulness of PFTs in quantitatively evaluating changes in lung function which might only be apparent after significant treatment time has been surpassed. Definitive DIPNECH guidelines stating a time frame for PFT measurement during follow-up would thus help reduce variance and improve comparability between studies.

The use of PFTs to objectively measure treatment response represents a meaningful improvement from previous studies which primarily reported subjective findings [18]. However, the correlation between changes in PFTs and representative changes in symptoms is not always equal. Generally, studies have shown that an improvement in PFTs corresponds with an improvement in symptoms [40]. However, in one study 20% of patients had normal PFTs at baseline despite reporting respiratory symptoms [24]. This is consistent with findings in COPD research where the term GOLD stage-0 is used to indicate symptomatic patients without lung function abnormalities [42]. PFTs may not be the complete measure for evaluating symptomatic improvements but nevertheless, remain an essential tool for clinicians managing chronic respiratory diseases.

Treatment success defined by an improvement in PFT following treatment is influenced by numerous factors. These include patient characteristics, the efficacy of treatment and the heterogeneity of DIPNECH between individuals. However, for treatment-related changes in PFT to manifest, a sufficient follow-up duration is needed. A 2020 study of 44 patients reported no significant improvements in PFT for half their SSA sample but the median follow-up time was only 30 months [51]. Short follow-up times may not accurately represent the time needed for PFT changes to appear especially in a heterogenous condition such as DIPNECH.

Surgical resection plays a common role in the initial management of suspected DIPNECH patients with dominant nodule(s). Resection aims to not only remove the dominant nodule(s) and thus reduce the progression of the disease, but also allows for histopathological analysis needed for a definitive diagnosis. In one study, nine patients underwent thoracotomy and resection of the dominant nodule prior to any initiation of pharmacological treatment [23]. The 2021 retrospective study also reported that 45 patients from their sample of 59 underwent initial wedge or lobe resections [52]. Studies outside of the DIPNECH literature have shown that surgical resection through lobectomy or video-assisted thoracic surgery (VATS) decreases pulmonary function in the short term, with some recovery of function to baseline noted on longer follow-up [53,54]. As both surgical resection and SSA treatment influence PFTs, the true lone impact of SSAs on PFTs becomes harder to determine. In cases with short-follow up times, any benefit to lung function from SSA treatment would be decreased by lag effects from earlier surgical resection. The abundance of nodules and tumourlets throughout the lung in a typical DIPNECH presentation renders curative surgical resection an inviable option, confined to resections of the dominant nodule as described above. The 2015 longitudinal clinical study discussed the use of transbronchial biopsy as a feasible alternative method for obtaining histopathological samples [7]. This could prove an increasingly popular option for patients whose risk profiles for surgery are higher or whose dominant nodules are positioned in more vascular areas.

Furthermore, treatment often comprised concomitant medications in addition to the SSAs. These included steroids, inhalers and narcotic cough medicines aimed at alleviating symptoms [23]. Their use reduces the ability to solely attribute changes in PFTs to SSA therapy. A future solution may involve a double-blind which would concretely represent the true effectiveness of SSAs.

## Conclusions

In this review, we have described how PFTs, and in particular spirometry, can be adopted from other areas of respiratory disease management and fundamentally incorporated into DIPNECH management. PFTs help evaluate treatment efficacy and are used to effectively monitor disease progression. Currently, SSA remains licenced for palliation of symptoms but the growing number of reports utilising these drugs as an initial treatment for DIPNECH coupled with evidence for their antiproliferative abilities influenced our choice of therapeutic. Evidence that SSTR expression increases with tumour differentiation corroborate with studies showing uptake accumulating within dominant nodules during SSTR imaging. Therefore, we can hypothesise that SSAs may have a greater effect on more advanced cases of DIPNECH where greater differentiation across the spectrum of pre-cancerous to cancerous cells are present or in cases where surgical resection of the dominant nodule was not possible. Nevertheless, incorporating PFTs as standard management protocol helps objectively monitor patients and thus allows clinicians to gather more data about the pathophysiology of DIPNECH and the efficacy of treatments.

Many studies are limited by sample size, owing to the rarity of the disease and the result of common misdiagnoses of patients. However, the clear acceleration in recognition of DIPNECH in recent years is hugely promising and will likely yield future targeted therapies that have the potential to change the current outlook. Whether SSAs will be included in these future therapies remains to be seen. In any case, the incorporation of PFTs into DIPNECH management represents a huge step forward allowing clinicians to accurately stratify patients and providing the opportunity for more personalised therapies.
